# Magnetic and luminescent hybrid nanomaterial based on Fe_3_O_4_ nanocrystals and GdPO_4_:Eu^3+^ nanoneedles

**DOI:** 10.1007/s11051-012-1188-7

**Published:** 2012-09-19

**Authors:** Marcin Runowski, Tomasz Grzyb, Stefan Lis

**Affiliations:** Department of Rare Earths, Faculty of Chemistry, Adam Mickiewicz University, Grunwaldzka 6, 60-780 Poznan, Poland

**Keywords:** Magnetic properties, Luminescence, Magnetite, Phosphates, Eu^3+^ doping, Nanoneedles

## Abstract

**Abstract:**

A bifunctional hybrid nanomaterial, which can show magnetic and luminescent properties, was obtained. A magnetic phase was synthesized as a core/shell type composite. Nanocrystalline magnetite, Fe_3_O_4_ was used as the core and was encapsulated in a silica shell. The luminescent phase was GdPO_4_ doped with Eu^3+^ ions, as the emitter. The investigated materials were synthesized using a coprecipitation method. Encapsulated Fe_3_O_4_ was “trapped” in a nano-scaffold composed of GdPO_4_ crystalline nanoneedles. When an external magnetic field was applied, this hybrid composite was attracted in one direction. Also, the luminescent phase can move simultaneously with magnetite due to a “trapping” effect. The structure and morphology of the obtained nanocomposites were examined with the use of transmission electron microscopy and X-ray powder diffraction. Spectroscopic properties of the Eu^3+^-doped nanomaterials were studied by measuring their excitation and emission spectra as well as their luminescence decay times.

**Graphical Abstract:**

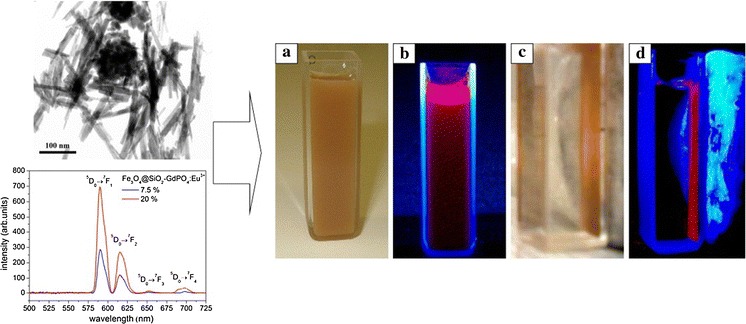

## Introduction

Nanocomposite structures with suitable dual or multifunctional properties have the potential to be useful in applications in areas such as biology and medicine (biomarkers, drug delivery, cells labeling, sensitive to magnetic field nanoparticles for the photodynamic therapy) and also in optoelectronics and lightening (Mi et al. 2010; Sun et al. 2010; Hu et al. 2011). Many research groups are currently focussed on the synthesis as well as the exploration of the properties of core/shell type nanostructures and other, similar composites like hybrid nanomaterials (Binnemans 2009; Dosev et al. 2007; Yu et al. 2006; Hu et al. 2010). Core/shell structures are usually composed of homogeneous nanosized particles as cores, which are wrapped by inert or active mono- or multishells. Examples of core/shell structures are metallic particles, which can be covered by silica shell (Liz-Marzán et al. 1996) or semiconductor nanoparticles like CdSe@SiO_2_ (Correa-Duarte et al. 1998). The composition of the cores and shells are not restricted, but the characteristics of shells have the greatest influence on the properties of the final product. Other factors which can potentially influence core/shell type compound properties are: the ratio of the number of particles in the core to the number of particles in the shell, particles size, homogeneity and distribution of their size, crystallinity and morphology, dopant concentrations, and impurities and defects in the structures.

Cores, which can show magnetic behavior, are composed of the oxides Fe_3_O_4_, Co_3_O_4_, or metal Ni, FePt nanoparticles (Runowski et al. 2011; Park et al. 2010; López Pérez et al. 1997; Son et al. 2007; Chen et al. 2011; Yano et al. 2009). Synthesis of core/shell structures can avoid the oxidation of cores (Sobal et al. 2002). Also, inert SiO_2_ nanoparticles are often used as cores (Yu et al. 2006). Other examples are semiconductors like ZnO and CdSe (Wang et al. 2010a; Darbandi et al. 2005) or inorganic, lanthanide-doped compounds like fluorides e.g., CeF_3_:Tb^3+^ and NaYF_4_:Yb^3+^,Er^3+^ (Runowski et al. 2011; Hu et al. 2011). The shell can also show fluorescent properties (organic or inorganic phosphors), e.g., rhodamine, LaF_3_:Ce^3+^,Tb^3+^, or CdTe (Sun et al. 2010; Chang et al. 2008; He et al. 2009). Functionalization of the shell can be accomplished by covering the surface of the particle with compounds which can behave as coordination ligands having appropriate groups, e.g., –SH and –NH_2_. Hydrophobic ligands on the surface of the as-prepared nanoparticles may be replaced by ligand-exchange reactions; employing one of a wide variety of currently used organic compounds like polyethylene glycol and its derivatives (PEG), polyacrylic acid (PPA) (Shenoy et al. 2006; Wang et al. 2010b), or polyethylenimine (PEI) (Wang et al. 2007). Alternatively, native capping ligands can be modified by oxidation or more advanced ligand engineering like polymerization or by reactions with additional compounds.

These hybrid structures may also be used in water purification from heavy metals, taking advantage of the large affinity of these composite particles, with large surface ratios, to the cations of metals, e.g., Pb^2+^, Cd^2+^, and Hg^2+^ (Wang et al. 2010c). This large affinity is due to the large surface area of nanostructures and to binding of the cations via –NH_2_ groups or via physical forces. When the above-mentioned structures are magnetic, an external magnetic field (like a strong magnet) can be used for very easy and quick purification of the structures. Another example of the potential application for nanohybrids, with magnetic cores, is in the area of condensing and removing bacteria and other microorganisms from water using the properties of appropriately biomodified shells. Core/shell structures and other advanced nanocomposites may also be used as biomarkers and contrast agents in imaging techniques like MRI (magnetic resonance imaging), which use paramagnetic or superparamagnetic compounds (usually based on gadolinium or iron oxide compounds) (Zhu et al. 2011).

When luminescent particles (organic compounds, polymers, quantum dots, rare earth-based UV-excited or upconversion phosphors) are incorporated into the shells, which could bind with microorganisms (binding with antibodies, antigens, polypeptides, etc.) or tumor tissue (Nyk et al. 2008; Selvan et al. 2009), the biocomplex can be localized by irradiation with UV or NIR light. After that due to luminescent and magnetic (optionally) properties, the biocomplex can be cured or removed. Also, drug delivery could be enhanced using magnetic nanoparticles (Yang et al. 2009).

When luminescent particles (organic compounds, polymers, quantum dots, rare earth-based UV-excited or upconversion phosphors) are incorporated into the shells, which could bind with microorganisms (binding with antibodies, antigens, polypeptides, etc.) or tumor tissue (Nyk et al. 2008; Selvan et al. 2009), the biocomplex can be localized by irradiation with UV or NIR light. Also, drug delivery could be enhanced using magnetic nanoparticles (Yang et al. 2009).

## Experimental

### Synthesis of magnetic core (Fe_3_O_4_)

The starting materials, 5.4 g FeCl_3_·6H_2_O (POCh S.A., pure p.a.) and 2.78 g FeSO_4_·7H_2_O (POCh S.A., pure p.a.) were mixed together in the molar ratio 2:1 and dissolved in distilled water. Subsequently, 0.5 g hydrazine sulfate (N_2_H_4_·H_2_O, Sigma-Aldrich, reagent grade) was added to the water solution as an antioxiding agent. The obtained solution was transferred to a round-bottom flask and filled with water up to 400 mL. Afterward an ammonia solution (20 mL of 10 % solution, POCh S.A., pure p.a.) was injected into the previous solution. Immediately, a black precipitate of Fe_3_O_4_ nanoparticles was obtained via the modified Massart method (Massart 1981). The reaction was continued for approximately 1 h. Intense stirring was maintained during the whole reaction process. The temperature of the solution was set to 50 °C. When the reaction process was finished, the solution was ultrasonicated for approximately 1 h, to produce well-dispersed, small, and homogeneous nanoparticles of magnetite. The obtained nanoparticles of Fe_3_O_4_ were separated using a strong magnet and washed several times with water.

### Synthesis of core/shell Fe_3_O_4_/SiO_2_

Covering the obtained Fe_3_O_4_ nanoparticles by a silica shell was accomplished using a modified Stöber method (Stöber et al. 1968). 0.03 g of the previously synthesized magnetite was dispersed in 50 mL of water and ultrasonicated for 30 min. The colloidal suspension of magnetite was then transferred to a beaker and filled with 25 mL of ethylene glycol (POCh S.A., pure p.a.) and 25 mL of glycerin (POCh S.A., pure p.a.). To the well-mixed homogeneous solution, 2.5 mL of tetraethyl orthosilicate (TEOS, Sigma-Aldrich, reagent grade, 98 %) was added. After that, 25 mL of the 10 % ammonia solution was added to start the hydrolysis process (the addition time was approximately 20 min.). After addition of ammonia, the reaction was continued for 50 min. The solution was intensely stirred and heated to 50 °C for the whole reaction process. The obtained core/shell nanoparticles of Fe_3_O_4_/SiO_2_ were separated with a strong magnet and washed several times with water and ethanol.

### Synthesis of the magnetic-luminescent nanocomposite based on Fe_3_O_4_/SiO_2_ and GdPO_4_:Eu^3+^ nanoneedles

The entire amount of the previously obtained Fe_3_O_4_/SiO_2_ nanoparticles was dispersed in a mixture of 50 mL water, 25 mL ethylene glycol, and 25 mL glycerin. Ammonium phosphate monobasic ((NH_4_)H_2_PO_4_, Sigma-Aldrich, ReagentPlus^®^, ≥98.5 %) was dissolved in the prepared solution and stirred together for 15 min. Subsequently the rare-earth nitrates, Gd(NO_3_)_3_ and Eu(NO_3_)_3_, were prepared by dissolving the oxides Gd_2_O_3_ and Eu_2_O_3_ (stanford materials, 99.99 %) in HNO_3_ (POCh S.A., ultra-pure). Obtained nitrates were dissolved in the system composed of water, ethylene glycol, and glycerin (in the ratio mentioned above). All the reactants were used in stoichiometric ratios, assuming the synthesis of 1 g of the product, and Eu^3+^ molar concentrations of 7.5 or 20 %. The obtained mixture was dropped into a colloidal solution containing ammonium phosphate and Fe_3_O_4_/SiO_2_ nanoparticles within 15 min. When the addition was finished, the reaction was continued for 20 min; for better incorporation of the precipitate on the surface of nanoparticles. The solution was intensely stirred and heated to 50 °C during the whole reaction process. The obtained nanoparticles of the magnetic product were separated using a strong magnet and washed several times with water and ethanol.

### Apparatus

XRD patterns were obtained with a Bruker AXS D8 Advance diffractometer in Bragg–Brentano geometry, with Cu K_α1_ radiation in the 2θ range from 6° to 60°. The joint committee on powder diffraction standards (JCPDS) database was used for the phase identification. The Scherrer equation was used for calculating average sizes of the crystallites (Scherrer 1918)]:

$$ D = \frac{0.9\lambda }{{\cos \theta \sqrt {\beta^{2} - \beta_{0}^{2} } }} $$

where *D* is the average grain size, λ is the X-ray wavelength, the factor 0.9 is characteristic for spherical objects, θ is the diffraction angle, and β is the full-width at half-maximum of an observed peak. TEM images were measured with a JEM 1200 EXII, JOEL transmission electron microscope, using an accelerating voltage of 80 kV.

An Hitachi F-7000 fluorescence spectrophotometer with a 150 W xenon lamp was used for the determination of the luminescence properties (excitation, emission, and lifetimes) of the samples at room temperature. The obtained spectra were corrected for instrumental response.

The presence of magnetic properties of the final product was confirmed with a strong applied external magnetic field (rare-earth magnet).

## Results and discussion

**Fig. 1:**
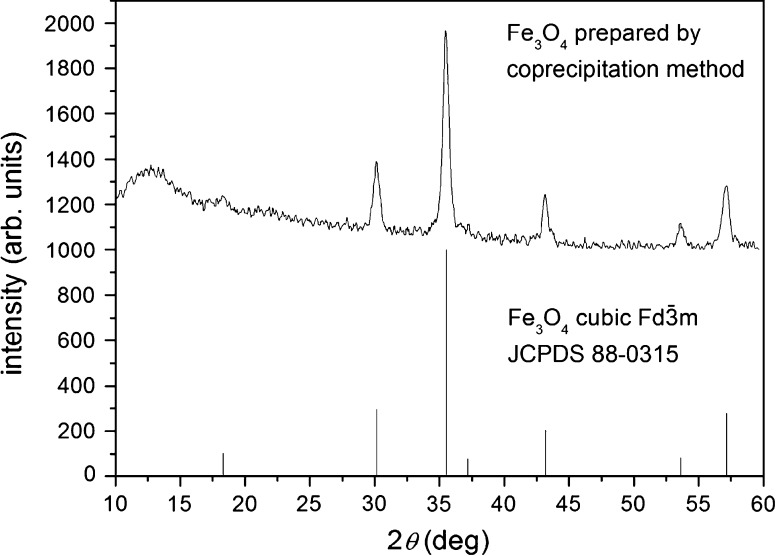
XRD pattern of nanocrystalline Fe_3_O_4_

A powder XRD analysis of the Fe_3_O_4_ nanoparticles (Fig. 1) confirmed the structure of magnetite. The estimated value of the average size of the magnetite particles from XRD, using the Scherrer equation, was approximately 17 ± 3 nm. The estimated value is in good correlation with the TEM image (Fig. 3).

**Fig. 2:**
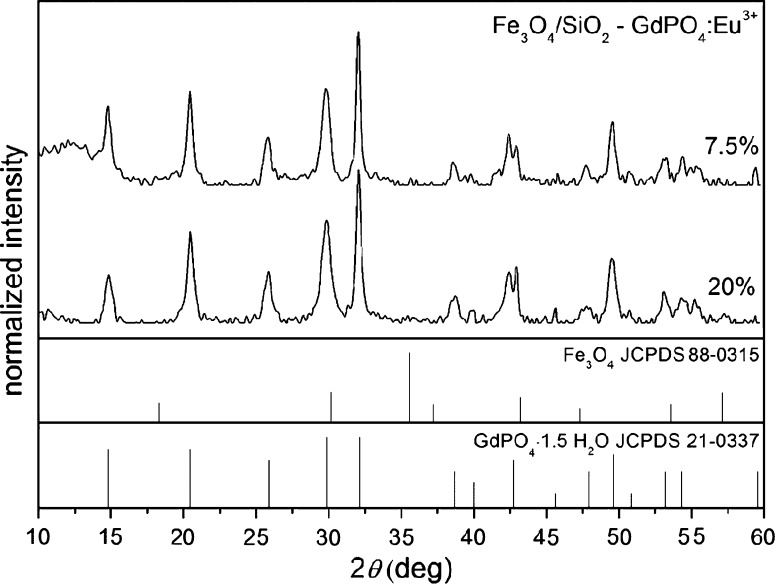
XRD patterns of hybrid, Fe_3_O_4_/SiO_2_–GdPO_4_:Eu^3+^ nanomaterials

The XRD patterns of the obtained hybrid nanomaterials are shown in Fig. 2. They are in good agreement with diffraction patterns from the database for the hydrated, hexagonal GdPO_4_·1.5H_2_O (JCPDS card no. 21-0337). Small changes in intensities and positions of some peaks were caused by the difference between the Gd^3+^ ion radius and the Eu^3+^ ion radius. Some reflection peaks originated from magnetite are almost invisible, due to the low concentration of Fe_3_O_4_ in total mass of the sample. There are no peaks from SiO_2_ because of the amorphous structure of the silica shell.

**Fig. 3:**
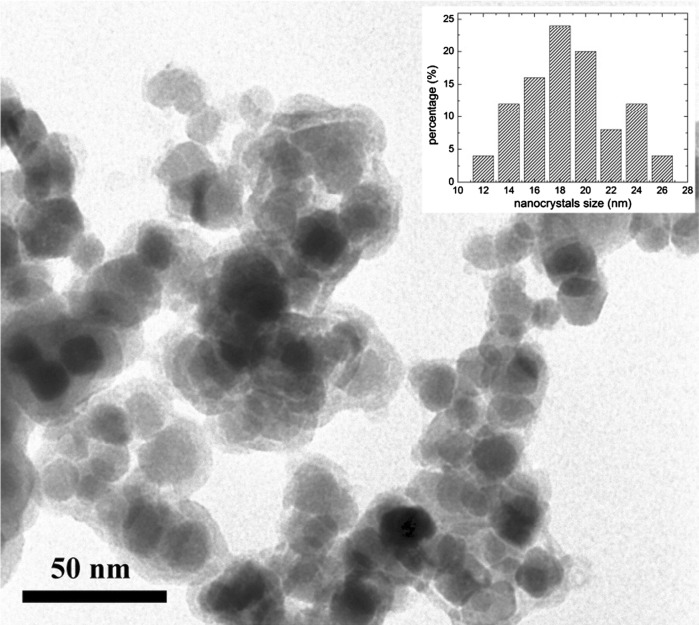
TEM image of core/shell Fe_3_O_4_/SiO_2_ nanoparticles and their size distribution

Figure 3 presents the TEM image of the core/shell Fe_3_O_4_/SiO_2_ nanostructure. In the picture, the formation of the core/shell type compound is clearly seen. The darker core corresponds to the magnetite, and the lighter shell is composed of the amorphous silica. The obtained core/shell type product showed magnetic behavior, which could be confirmed when an external magnetic field was applied. The size distribution of the nanoparticles was calculated using TEM data (Fig. 3). The relatively uniform size distribution of the grains is quite encouraging in our composite product.

**Fig. 4:**
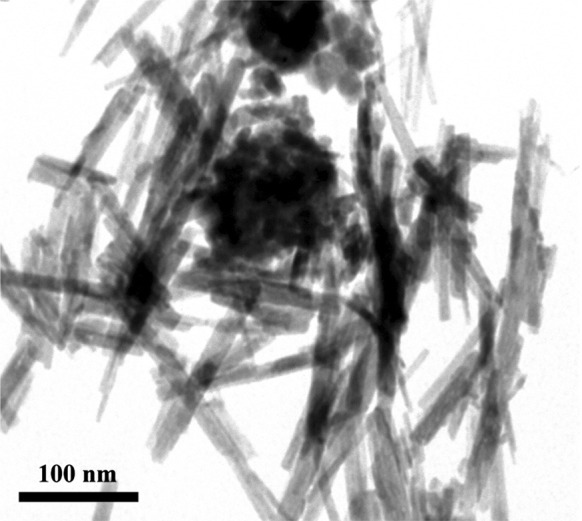
TEM image of nanocomposite based on Fe_3_O_4_/SiO_2_–GdPO_4_:Eu^3+^ 20 % nanoneedles

Figure 4 presents the TEM image of the composite, magnetic-luminescent nanomaterial based on Fe_3_O_4_/SiO_2_ and GdPO_4_:Eu^3+^ nanoneedles. The calculated dimensions (from the TEM picture) of these nanoneedles are 40–250 nm long and 3–15 nm wide. This complex nanocomposite material showed simultaneously magnetic and luminescent properties.

**Fig. 5:**
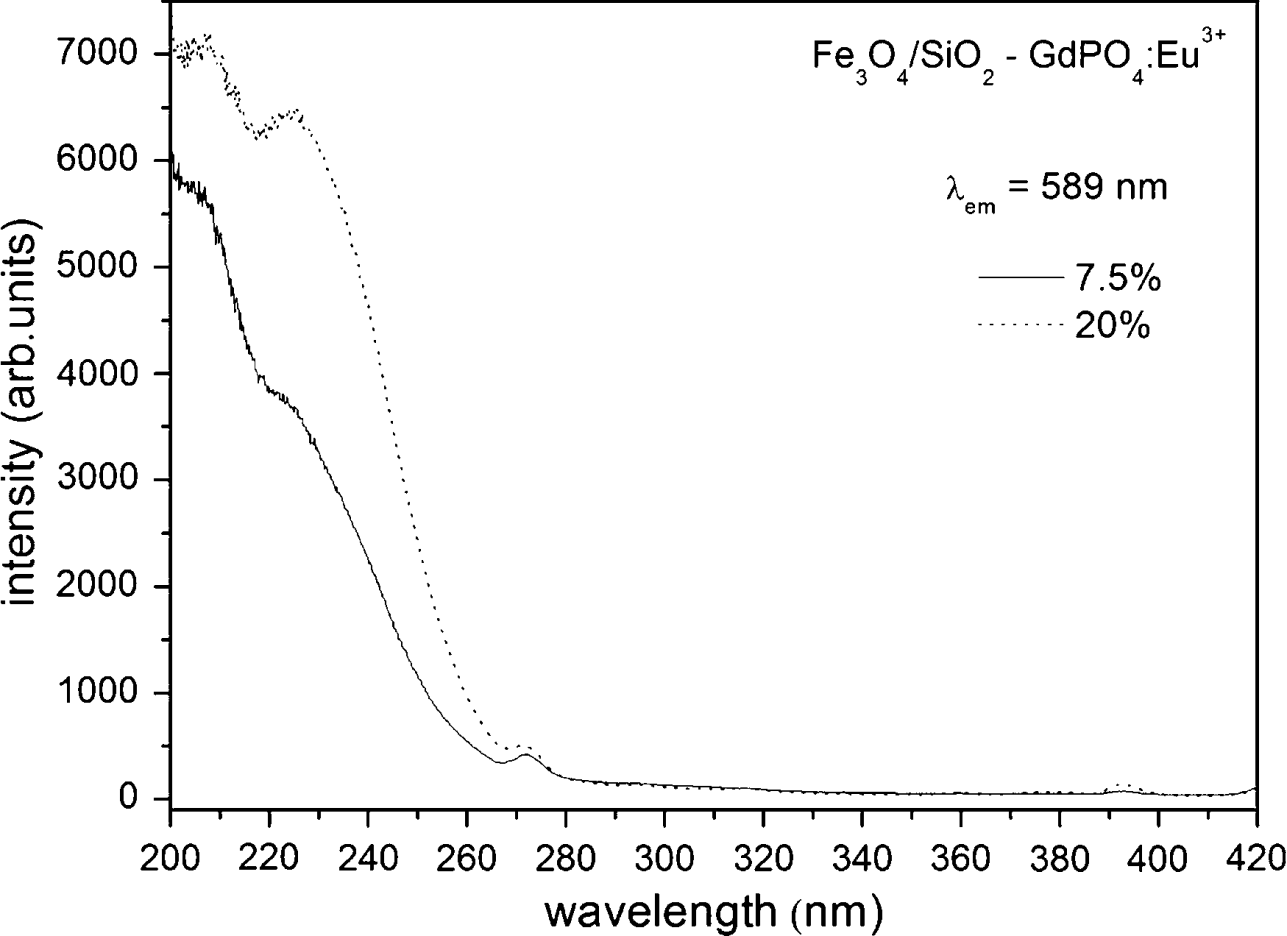
Excitation spectra of the Fe_3_O_4_/SiO_2_–GdPO_4_:Eu^3+^ hybrid material registered at room temperature

**Fig. 6:**
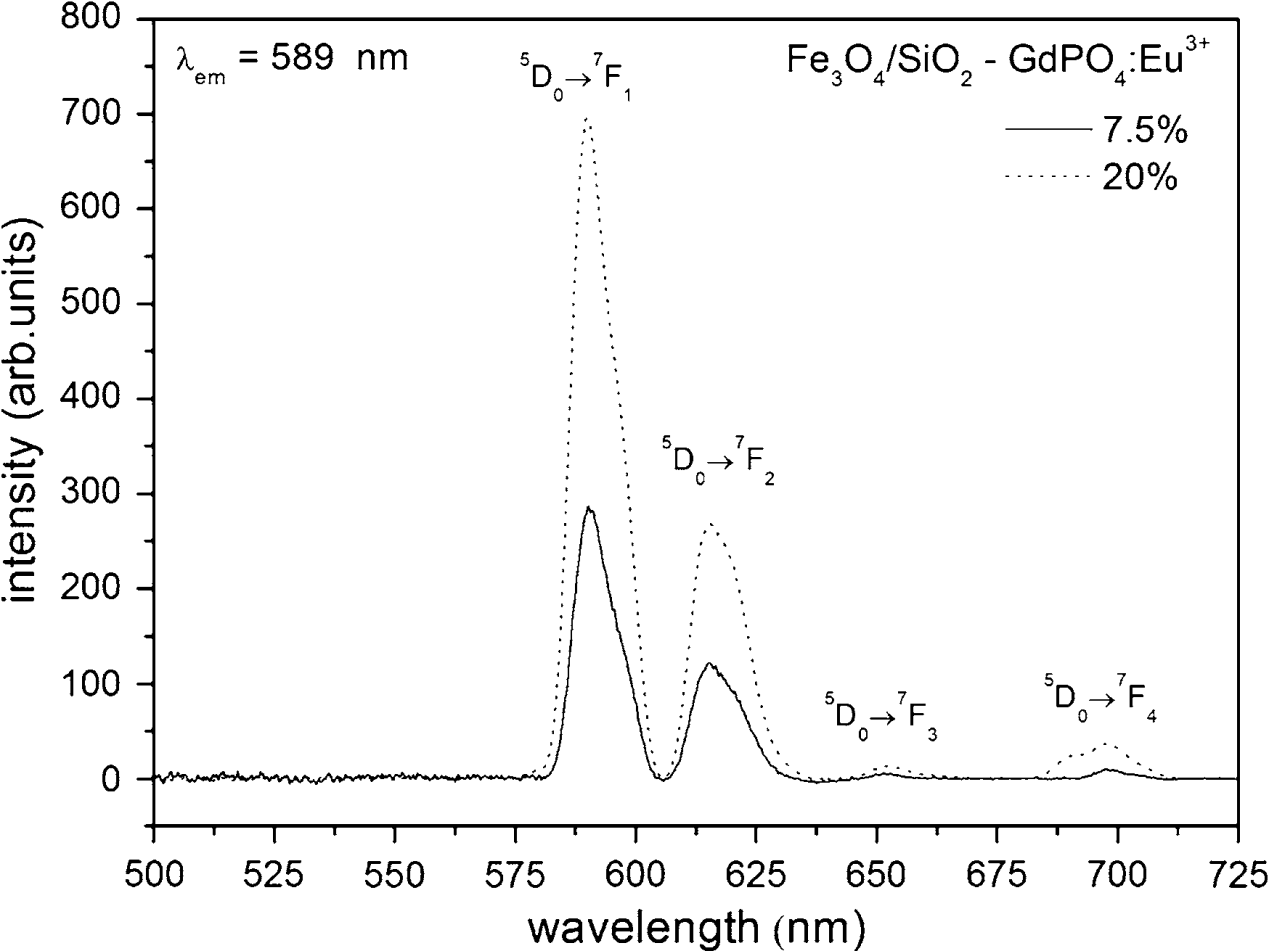
Emission spectra of the Fe_3_O_4_/SiO_2_–GdPO_4_:Eu^3+^ hybrid material registered at room temperature

Excitation and emission spectra are shown in Figs. 5, 6. Two concentrations of the dopant, Eu^3+^ ions, were used as emission activators. The prepared compound can be excited by UV radiation at the optimal wavelength of λ_ex_ = 245 nm. This wavelength corresponds to the maximum of the wide band, which is connected with charge transfer between the O^2−^ and Eu^3+^ ions. Less intense peaks around 270 and 390 nm are due to the energy transfer from Gd^3+^ to Eu^3+^ and to the f–f transitions in the Eu^3+^ ions, respectively.

When the concentration of Eu^3+^ was increased from 7.5 to 20 %, a more intense emission could be observed. Usually, the optimal concentration of a dopant-like Eu^3+^ in phosphors is in the range of 5–10 %. When the concentration is increased, cross-relaxation is observed, and the excited Eu^3+^ ions are quenched (Weber 1968). The observed small quenching in the higher doped sample could be connected with the abnormal environment of the Eu^3+^ ions. The shape of the GdPO_4_ nanoparticles (nanoneedles) results in a larger surface to volume ratio, compared to spherical objects. In such a case, the Eu^3+^ ions are mostly on the surface. Therefore, concentration quenching is less efficient, and the optimal concentration is shifted.

The observed emission was intensely red, and four transition peaks characteristic for the Eu^3+^ emission spectrum were observed. The ratio of ^5^D_0_ → ^7^F_1_ to ^5^D_0_ → ^7^F_2_ transitions is characteristic of the high-symmetry environment of the Eu^3+^ ions (Görller-Warland and Binnemans 1998). Two low-energy transitions ^5^D_0_ → ^7^F_3_ and ^5^D_0_ → ^7^F_4_ were also observed.

**Fig. 7:**
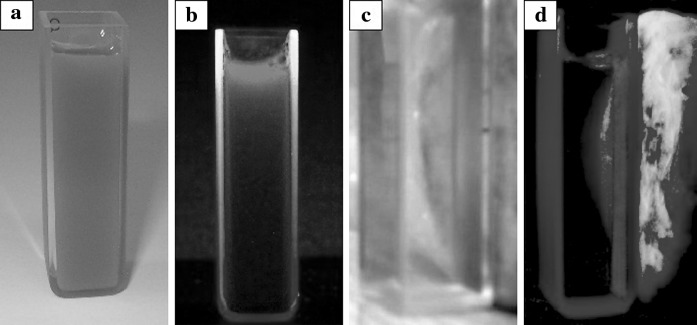
Luminescent and magnetic properties of bifunctional nanocomposite (20 % Eu^3+^), before (**a**, **b**) and after (**c**, **d**) magnet capture. Shown samples were irradiated by UV lamp (λ = 254 nm)

Figure 7 presents the luminescent and magnetic properties of the Fe_3_O_4_/SiO_2_–GdPO_4_:Eu^3+^ 20 % nanoproduct dispersed in water. It is clearly seen that, after magnet capture, all the nanoparticles were attracted to the cuvette wall. When the UV light was on, the characteristic red luminescence of the Eu^3+^ ion was observed. This behavior confirms the formation of the hybrid, bifunctional nanophosphor.

**Fig. 8:**
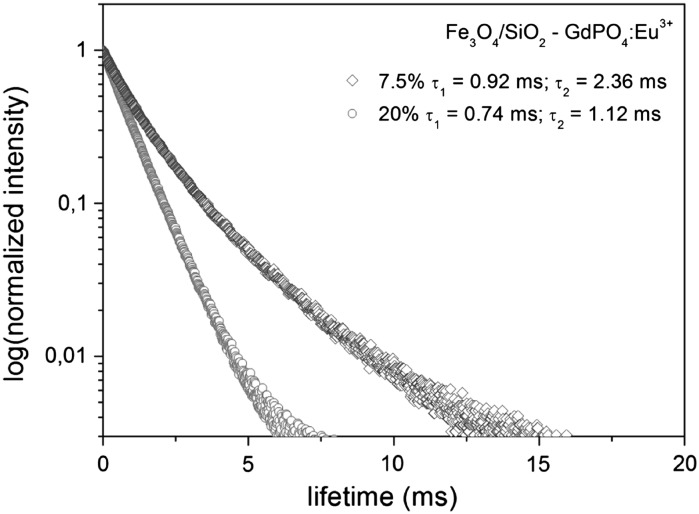
Luminescence decays of Eu^3+^ in Fe_3_O_4_/SiO_2_–GdPO_4_:Eu^3+^ observed at λ_em_ = 589 nm and excited at λ_ex_ = 245 nm

Emission decay curves of the prepared materials are presented in Fig. 8. The obtained curves were both fitted to biexponential decays with good correlation coefficients. The two lifetimes originate from two different Eu^3+^ ions, occupying sites inside the nanocrystals and on their surface, where the ions are surrounded by different environments. Their values are typical for Eu^3+^ in inorganic hosts (Liu et al. 2007; Wiglusz et al. 2010), and both are shortened in samples with higher concentrations of Eu^3+^. The shorter lifetimes are due to the concentration quenching.

## Conclusions

A bifunctional, magnetic-luminescent, nanomaterial was obtained, using a simple, coprecipitation method. The described nanocomposite is sensitive to external magnetic fields. The obtained product was attracted to magnets both in the solid state (as a dry powder) and in water dispersed state as a colloid. The UV lamp with a maximum emission at 254 nm was used to excite this composite, and an intense red luminescence was visible. Such a luminescence was also observable in an aqueous system, which is not common for Eu^3+^ containing compounds. These ions are usually quenched in aqueous medium due to vibronic coupling with O–H oscillators (Lis 2002; Klonkowski et al. 2003). We report that quenching was minimal in the synthesized nanocomposite. This property can be of interest in potential applications. However, the mechanism of this phenomena and the influence of the environment should be examined and discussed in further research.

The properties of the discussed nanocomposite were examined using photophysical methods. This nanohybrid product showed simultaneously magnetic and luminescent properties and can be used in many fields of science (e.g., nanochemistry, biochemistry, pharmacy, medicine, and industry). These modern biochemical and physicochemical approaches based on nanotechnology could be enhanced due to the novel properties of these nanomaterials.
